# Crystal structure of MgK_0.5_[B_6_O_10_](OH)_0.5_·0.5H_2_O, poly[dimagnesium potassium bis(hexa­borate) hy­droxide monohydrate]

**DOI:** 10.1107/S2056989022008982

**Published:** 2022-09-08

**Authors:** Qi-Ming Qiu, Jian-Biao Song

**Affiliations:** a School of Science, China University of Geosciences, Beijing 100083, People’s Republic of China; b Beijing Chaoyang Foreign Language School, Beijing 100101, People’s Republic of China; University of Neuchâtel, Switzerland

**Keywords:** alkali–earth-metal borate, alkaline-earth-metal borate, solvothermal synthesis, three-dimensional framework, crystal structure

## Abstract

The solvothermal reaction of H_3_BO_3_, KCF_3_SO_3_, Mg(CF_3_SO_3_)_2_ and pyridine led to a new alkali- and alkaline-earth-metal borate, MgK_0.5_[B_6_O_10_](OH)_0.5_·0.5H_2_O. Its structure features an intricate three-dimensional framework built from [B_6_O_13_]^8−^ clusters, thus resulting in a six-connected achiral net with high symmetry. Each [B_6_O_13_]^8−^ building block is composed of three trigonal BO_3_ and three tetra­hedral BO_4_ units, with these BO_4_ units being further connected to neighboring BO_3_ units, giving rise to an oxoboron cluster of the general formula [B_6_O_10_]^2−^.

## Abstract 2

The solvothermal reaction of H_3_BO_3_, KCF_3_SO_3_, Mg(CF_3_SO_3_)_2_ and pyridine led to a new alkali- and alkaline-earth-metal borate. Its structure features an intricate three-dimensional framework built from [B_6_O_13_]^8−^ clusters, thus resulting in a six-connected achiral net with high symmetry.

## 1. Chemical context

As inorganic materials, borates are an important class of non-linear optical crystals, mainly because they can easily crystallize in non-centrosymmetric space groups and such structures often show a large second-harmonic generation response (Qiu *et al.*, 2021*a* ▸; Qui & Yang, 2021*a* ▸). The combination of BO_3_-trigonal and BO_4_-tetra­hedral units makes it possible to form a variety of isolated anionic clusters. Extended chains, layers and three-dimensional frameworks can be formed between clusters through the dehydration and condensation of the terminal hydroxyl groups of oxoboron clusters (Wang *et al.*, 2017 ▸). In addition, negatively charged oxoboron clusters can also combine with a variety of counter-cations, making the structure of borates more complex and diverse. Here, single crystals of MgK_0.5_[B_6_O_10_](OH)_0.5_·0.5H_2_O with alkali- and alkaline-earth metals have been obtained under solvothermal conditions. 
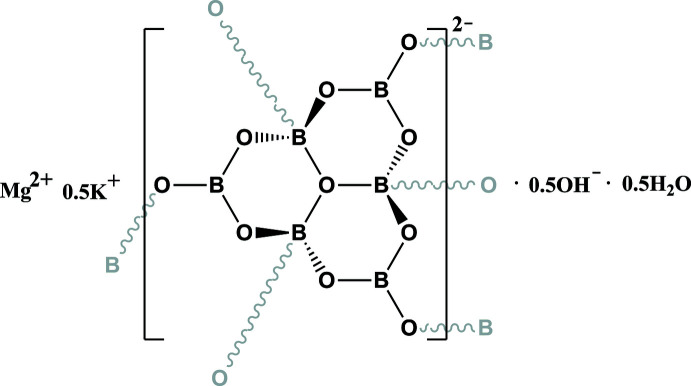


## 2. Structural commentary

**Figure 1:**
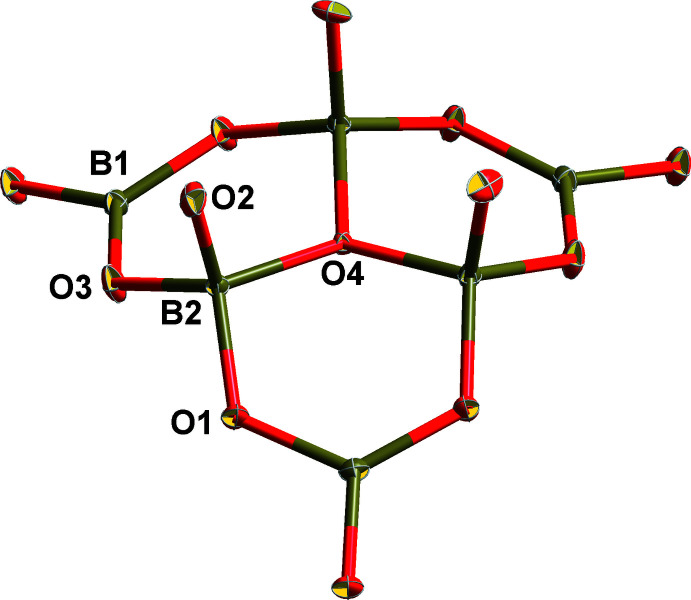
The asymmetric unit of the [B_6_O_13_]^8−^ oxoboron cluster. Displacement ellipsoids are drawn at the 50% probability level.

**Figure 2:**
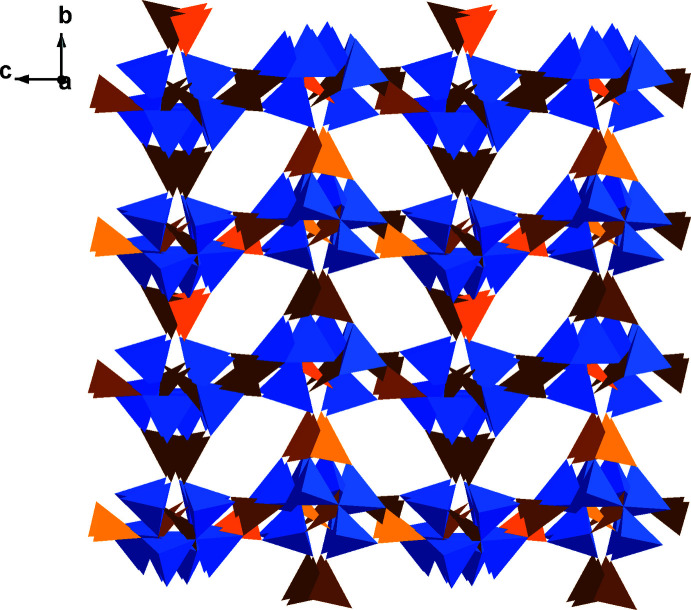
View of the three-dimensional supra­molecular framework along the [100] direction. Color code: BO_3_ trigonal, yellow, orange and brown; BO_4_ tetra­hedral, blue.

The asymmetric unit of the title compound consists of 2 B, 10/3 O, 1/3 Mg, 1/6 K, 1/6 OH, and 1/6 H_2_O. The Mg, K, O4, O5 and O6 atoms are located on special positions with occupancy of 1/3 or 1/6, while the remaining B and O atoms are located at general positions with an occupancy of 1. Bond-valence-sum calculations show that Mg, K and B are consistent with the expected oxidation states (Brown & Altermatt, 1985 ▸; Brese & O’Keeffe, 1991 ▸). Three BO_4_ units are joined together through corner-sharing of the O4 atom and three BO_4_ units are connected with three neighboring BO_3_ units to form a [B_6_O_13_]^8−^ oxoboron cluster (Fig. 1 ▸). To the best of our knowledge, this is the first example of a mixed alkali- and alkaline-earth-metal borate crystal with the [B_6_O_13_]^8−^ cluster anion. In this cluster, the B—O4 bonds are unique because their bond distances [1.529 (2) Å] are longer than other B—O bonds [1.359 (2)–1.453 (2) Å] in the BO_3_ and BO_4_ units. Each [B_6_O_13_]^8−^ unit is further connected to six other clusters by corner-sharing O atoms, resulting in a three-dimensional framework (Fig. 2 ▸).

## 3. Supra­molecular features

In the title compound, the Mg and K atoms are six-coord­inated, with Mg—O and distances in the range 2.332 (1)–2.374 (1) Å and K—O = 2.845 (1) Å. The three-dimensional structure is stabilized by a water cluster formed by O5—H5⋯O5, O5—H5⋯O6 and O6—H6*A*⋯O2 hydrogen bonds involving the water mol­ecule, hydroxyl group and oxoboron cluster (Table 1 ▸). The channels of the compound are filled with ions/mol­ecules (Mg^2+^, K^+^, OH^−^ and H_2_O). The title structure is similar to previously reported analogues NH_4_NaB_6_O_10_ (Wang *et al.*, 2014 ▸), K_0.5_[B_6_O_10_]·H_2_O·1.5H_3_O (Qiu & Yang, 2021*b* ▸), and NaRb_0.5_[B_6_O_10_]·0.5H_3_O (Qiu *et al.*, 2021*b* ▸), so the simultaneous use of NH_4_ and Na or K or Na and Rb or Mg and K cations as templates has no effect on the crystallization of the oxoboron three-dimensional framework. However, after the introduction of Cl (Wu *et al.*, 2011 ▸) or Br (Al-Ama *et al.*, 2006 ▸), the new compounds crystallize in the trigonal space group *R*3*m* with a large second-harmonic generation response. The introduction of different anions can therefore play a key role in changing the crystalline structure to a non-centrosymmetric system.

## 4. Database survey

A search of the Cambridge Structural Database (CSD, version 5.43, update June 2022; Groom *et al.*, 2016 ▸) for the [B_6_O_13_]^8−^ oxoboron cluster gave 23 hits. The terminal oxygen atoms of this type of [B_6_O_*x*_] unit can be completely deprotonated [B_6_O_13_]^8−^, partially protonated [B_6_O_11_(OH)_2_]^6−^ or completely protonated [B_6_O_7_(OH)_6_]^2−^. Among the above 23 compounds, most of them are inorganic–organic hybrid solids, which contain transition-metal complexes and the [B_6_O_7_(OH)_6_]^2−^ cluster (refcodes: CAFYIV, CAFYOB, Altahan *et al.*, 2021 ▸; CECWEM, Heller & Schellhaas, 1983 ▸; EMEHIP, Li *et al.*, 2016 ▸; HIXNAF, Jamai *et al.*, 2014 ▸; JOCCUC, JOCDAJ, Altahan *et al.*, 2019*a* ▸; JUZLIC, Altahan *et al.*, 2020 ▸; MEBQUI, MEBRET, Altahan *et al.*, 2017 ▸; POJVIW, POJVOC, Altahan *et al.*, 2019*b* ▸; TAFROI, Natarajan *et al.*, 2003 ▸; VUVLOP, Jemai *et al.*, 2015 ▸; BATCUY, Jamai *et al.*, 2022 ▸; SAZVEY, Xin *et al.*, 2022 ▸). It is worth noting that this oxoboron cluster contains too many active hydroxyl groups and therefore tends to form isolated structures. In the crystal of [Cd(1,2-dap)]·[B_6_O_11_(OH)_2_]·H_2_O (1,2-dap = 1,2-di­amino­propane, refcode: LOZZUY, Deng *et al.*, 2020 ▸) and Cd_3_[B_6_O_9_(OH)_2_]_2_·2NO_3_·4H_2_O (refcode: ZUXLIQ, He *et al.*, 2020 ▸), partially protonated [B_6_O_11_(OH)_2_]^6−^ was successfully extended to layered structures *via* B—O—B bonds. In the crystal of NaRb_0.5_[B_6_O_10_]·0.5H_3_O (refcode: UCEXOT, Qiu *et al.*, 2021*b* ▸), each completely deprotonated [B_6_O_13_]^8−^ unit was linked to six nearest neighbors by bridging O atoms, leading to a 3D framework, similar to that of the title compound.

## 5. Synthesis and crystallization

A mixture of H_3_BO_3_ (0.618 g, 10 mmol), KCF_3_SO_3_ (0.188 g, 1 mmol) and Mg(CF_3_SO_3_)_2_ (0.322 g, 1 mmol) was added to pyridine (3.0 mL). After stirring for 20 min, the resulting mixture was sealed in a 25 mL Teflon-lined stainless steel autoclave, heated at 488 K for 9 d, and then slowly cooled to room temperature and colorless block-shaped crystals MgK_0.5_[B_6_O_10_](OH)_0.5_·0.5H_2_O were obtained (yield 56% based on H_3_BO_3_). Infrared (KBr pallet, cm^−1^): 3190*vs*, 1631*s*, 1360*s*, 1268*m*, 1188*m*, 1134*m*, 1099*m*, 964*s*, 845*m*, 781*m*, 741*m*, 718*m*, 630*w*, 564*w*, 540*w*, 480*w*, 455*w*.

## 6. Refinement

Crystal data, data collection and structure refinement details are summarized in Table 2 ▸. Hydrogen atoms were positioned geometrically (O—H = 0.85 Å) and refined as riding with *U*_iso_(H) 1.2*U*_eq_(O).

## Supplementary Material

Crystal structure: contains datablock(s) I, global. DOI: 10.1107/S2056989022008982/tx2057sup1.cif

Structure factors: contains datablock(s) I. DOI: 10.1107/S2056989022008982/tx2057Isup2.hkl

CCDC reference: 2205808

Additional supporting information:  crystallographic information; 3D view; checkCIF report

## Figures and Tables

**Table 1:** Hydrogen-bond geometry (Å, °)

*D*—H⋯*A*	*D*—H	H⋯*A*	*D*⋯*A*	*D*—H⋯*A*
O5—H5⋯O5^i^	0.85	1.67	2.42 (3)	145
O6—H6*A*⋯O2^i^	0.85	2.58	3.276 (15)	140
O5—H5⋯O6^i^	0.85	1.78	2.484 (19)	139
O5—H5⋯O5^ii^	0.85	2.30	3.06 (3)	150

Symmetry codes: (i) 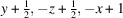 ; (ii) 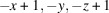 .

**Table 2:** Experimental details

Crystal data	
Chemical formula	Mg_2_K[B_6_O_10_]_2_(OH)·H_2_O
*M*_r_	572.46
Crystal system, space group	Cubic, *P* *a* 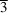
Temperature (K)	296
*a* (Å)	12.2966 (2)
*V* (Å^3^)	1859.32 (9)
*Z*	4
Radiation type	Mo *K*α
μ (mm^−1^)	0.47
Crystal size (mm)	0.10 × 0.08 × 0.08

Data collection	
Diffractometer	Bruker APEXII CCD
Absorption correction	Multi-scan (*SADABS*; Krause et al., 2015 ▸)
*T*_min_, *T*_max_	0.762, 0.936
No. of measured, independent and observed [*I* > 2σ(*I*)] reflections	23808, 952, 828
*R*_int_	0.056
(sin θ/λ)_max_ (Å^−1^)	0.714

Refinement	
*R*[*F* ^2^ > 2σ(*F* ^2^)], *wR*(*F* ^2^), *S*	0.039, 0.116, 1.16
No. of reflections	952
No. of parameters	72
H-atom treatment	H-atom parameters constrained
Δρ_max_, Δρ_min_ (e Å^−3^)	0.66, −0.64

Computer programs: *APEX2* and *SAINT* (Bruker, 2006 ▸), *SHELXT2018/3* (Sheldrick, 2015*a* ▸), *SHELXL2018/3* (Sheldrick, 2015*b* ▸), and *SHELXTL* (Sheldrick, 2008 ▸).

## supplementary crystallographic information

### Crystal data

**Table:**

MgK_0.5_[B_6_O_10_](OH)_0.5_·0.5H_2_O	Mo *K*α radiation, λ = 0.71073 Å
*M**_r_* = 572.46	Cell parameters from 5324 reflections
Cubic, *P**a*3	θ = 2.9–30.3°
*a* = 12.2966 (2) Å	µ = 0.47 mm^−^^1^
*V* = 1859.32 (9) Å^3^	*T* = 296 K
*Z* = 4	Block, colorless
*F*(000) = 1128	0.10 × 0.08 × 0.08 mm
*D*_x_ = 2.045 Mg m^−^^3^	

### Data collection

**Table:**

Bruker APEXII CCD diffractometer	828 reflections with *I* > 2σ(*I*)
Radiation source: fine-focus sealed tube, Bruker (Mo) X-ray Source	*R*_int_ = 0.056
φ and ω scans	θ_max_ = 30.5°, θ_min_ = 3.3°
Absorption correction: multi-scan (SADABS; Krause et al., 2015)	*h* = −16→17
*T*_min_ = 0.762, *T*_max_ = 0.936	*k* = −16→16
23808 measured reflections	*l* = −16→17
952 independent reflections	

### Refinement

**Table:**

Refinement on *F*^2^	0 restraints
Least-squares matrix: full	Hydrogen site location: difference Fourier map
*R*[*F*^2^ > 2σ(*F*^2^)] = 0.039	H-atom parameters constrained
*wR*(*F*^2^) = 0.116	*w* = 1/[σ^2^(*F*_o_^2^) + (0.0658*P*)^2^ + 0.9552*P*] where *P* = (*F*_o_^2^ + 2*F*_c_^2^)/3
*S* = 1.16	(Δ/σ)_max_ < 0.001
952 reflections	Δρ_max_ = 0.66 e Å^−^^3^
72 parameters	Δρ_min_ = −0.64 e Å^−^^3^

### Special details

**Table:**

Geometry. All esds (except the esd in the dihedral angle between two l.s. planes) are estimated using the full covariance matrix. The cell esds are taken into account individually in the estimation of esds in distances, angles and torsion angles; correlations between esds in cell parameters are only used when they are defined by crystal symmetry. An approximate (isotropic) treatment of cell esds is used for estimating esds involving l.s. planes.
Refinement. The structure was solved by direct methods and refined by the full-matrix least-squares method on *F*^2^ using the SHELXL programs (Bruker, 2006; Sheldrick, 2015*a*). All non-hydrogen atoms in the complex were refined anisotropically.

### Fractional atomic coordinates and isotropic or equivalent isotropic displacement parameters (Å^2^)

**Table:**

	*x*	*y*	*z*	*U*_iso_*/*U*_eq_	Occ. (<1)
Mg	0.33884 (5)	0.33884 (5)	0.33884 (5)	0.0205 (3)	
K	0.500000	0.500000	0.500000	0.0257 (3)	
O1	0.52065 (8)	0.28854 (8)	0.29782 (8)	0.0110 (3)	
O2	0.22982 (9)	0.18931 (8)	0.38027 (8)	0.0118 (3)	
O3	0.36386 (8)	0.68000 (8)	0.55091 (8)	0.0113 (3)	
O4	0.18887 (7)	0.18887 (7)	0.18887 (7)	0.0057 (3)	
O5	0.4745 (13)	0.1216 (14)	0.5078 (12)	0.066 (4)	0.1667
H5	0.504419	0.061354	0.523614	0.099*	0.1667
O6	0.544 (2)	0.0524 (17)	0.5575 (10)	0.080 (6)	0.1667
H6A	0.548070	0.065436	0.625259	0.121*	0.1667
H6B	0.587560	0.097246	0.528419	0.121*	0.1667
B1	0.21526 (12)	0.22026 (12)	0.48534 (12)	0.0084 (3)	
B2	0.16682 (11)	0.13303 (11)	0.29771 (11)	0.0067 (3)	

### Atomic displacement parameters (Å^2^)

**Table:**

	*U*^11^	*U*^22^	*U*^33^	*U*^12^	*U*^13^	*U*^23^
Mg	0.0205 (3)	0.0205 (3)	0.0205 (3)	0.0007 (2)	0.0007 (2)	0.0007 (2)
K	0.0257 (3)	0.0257 (3)	0.0257 (3)	0.0071 (2)	0.0071 (2)	0.0071 (2)
O1	0.0060 (4)	0.0113 (5)	0.0156 (5)	0.0015 (3)	−0.0031 (3)	−0.0047 (4)
O2	0.0141 (5)	0.0157 (5)	0.0055 (4)	−0.0039 (4)	0.0005 (3)	−0.0023 (3)
O3	0.0138 (5)	0.0134 (5)	0.0066 (4)	0.0060 (4)	−0.0017 (4)	−0.0027 (3)
O4	0.0057 (3)	0.0057 (3)	0.0057 (3)	0.0007 (3)	0.0007 (3)	0.0007 (3)
O5	0.058 (8)	0.074 (11)	0.067 (9)	0.005 (7)	0.011 (7)	−0.018 (8)
O6	0.123 (19)	0.101 (16)	0.017 (5)	0.029 (12)	−0.018 (7)	−0.002 (6)
B1	0.0094 (6)	0.0091 (6)	0.0065 (6)	0.0007 (5)	−0.0008 (5)	−0.0009 (5)
B2	0.0067 (6)	0.0067 (6)	0.0067 (6)	0.0003 (4)	0.0006 (4)	0.0005 (5)

### Geometric parameters (Å, º)

**Table:**

Mg—O2^i^	2.3319 (12)	O2—B1	1.3587 (16)
Mg—O2^ii^	2.3319 (12)	O2—B2	1.4525 (16)
Mg—O2	2.3319 (12)	O3—B1^vii^	1.3570 (18)
Mg—O1^i^	2.3738 (10)	O3—B2^viii^	1.4519 (16)
Mg—O1^ii^	2.3738 (10)	O4—B2^i^	1.5285 (15)
Mg—O1	2.3738 (10)	O4—B2^ii^	1.5285 (15)
Mg—B1	2.7714 (15)	O4—B2	1.5285 (15)
Mg—B1^i^	2.7715 (15)	O5—O6^ix^	0.96 (2)
Mg—B1^ii^	2.7715 (15)	O5—O6^x^	1.27 (3)
Mg—K	3.4324 (11)	O5—O6	1.35 (3)
K—O3	2.8450 (10)	O5—H5	0.8500
K—O3^iii^	2.8450 (10)	O5—H6B	1.4451
K—O3^i^	2.8450 (10)	O5—H6A^ix^	0.70 (3)
K—O3^iv^	2.8450 (10)	O6—O6^ix^	1.20 (3)
K—O3^v^	2.8450 (10)	O6—O6^xi^	1.20 (3)
K—O3^ii^	2.8450 (10)	O6—H5	0.6476
O1—B1^ii^	1.3821 (18)	O6—H6A	0.8500
O1—B2^vi^	1.4531 (16)	O6—H6B	0.8500
			
O2^i^—Mg—O2^ii^	81.06 (5)	B1^ii^—O1—B2^vi^	123.77 (11)
O2^i^—Mg—O2	81.06 (5)	B1^ii^—O1—Mg	91.19 (8)
O2^ii^—Mg—O2	81.06 (5)	B2^vi^—O1—Mg	144.28 (8)
O2^i^—Mg—O1^i^	112.48 (4)	B1—O2—B2	136.70 (11)
O2^ii^—Mg—O1^i^	132.64 (4)	B1—O2—Mg	93.59 (8)
O2—Mg—O1^i^	58.46 (3)	B2—O2—Mg	121.98 (8)
O2^i^—Mg—O1^ii^	58.46 (3)	B1^vii^—O3—B2^viii^	122.23 (11)
O2^ii^—Mg—O1^ii^	112.48 (4)	B1^vii^—O3—K	125.09 (8)
O2—Mg—O1^ii^	132.64 (4)	B2^viii^—O3—K	110.32 (7)
O1^i^—Mg—O1^ii^	112.97 (3)	B2^i^—O4—B2^ii^	117.97 (4)
O2^i^—Mg—O1	132.64 (4)	B2^i^—O4—B2	117.97 (4)
O2^ii^—Mg—O1	58.46 (3)	B2^ii^—O4—B2	117.97 (4)
O2—Mg—O1	112.48 (4)	O6^ix^—O5—O6	59 (2)
O1^i^—Mg—O1	112.96 (3)	O6^x^—O5—O6	89.1 (19)
O1^ii^—Mg—O1	112.96 (3)	O6^ix^—O5—H5	47.9
O2^i^—Mg—B1	93.22 (5)	O6^x^—O5—H5	67.1
O2^ii^—Mg—B1	109.26 (5)	O6—O5—H5	22.1
O2—Mg—B1	29.29 (4)	O6^ix^—O5—H6B	94.6
O1^i^—Mg—B1	29.91 (4)	O6^x^—O5—H6B	104.1
O1^ii^—Mg—B1	123.25 (4)	O6—O5—H6B	35.2
O1—Mg—B1	121.16 (4)	H5—O5—H6B	50.4
O2^i^—Mg—B1^i^	29.30 (4)	O5^xi^—O6—O6^ix^	71 (3)
O2^ii^—Mg—B1^i^	93.22 (5)	O5^xi^—O6—O6^xi^	77 (3)
O2—Mg—B1^i^	109.26 (5)	O6^ix^—O6—O6^xi^	101 (2)
O1^i^—Mg—B1^i^	121.16 (4)	O5^xi^—O6—O5^xii^	114 (2)
O1^ii^—Mg—B1^i^	29.90 (4)	O6^ix^—O6—O5^xii^	136.9 (16)
O1—Mg—B1^i^	123.25 (4)	O6^xi^—O6—O5^xii^	45.7 (12)
B1—Mg—B1^i^	114.20 (3)	O5^xi^—O6—O5	107 (3)
O2^i^—Mg—B1^ii^	109.26 (5)	O6^ix^—O6—O5	43.9 (9)
O2^ii^—Mg—B1^ii^	29.30 (4)	O6^xi^—O6—O5	133.5 (16)
O2—Mg—B1^ii^	93.22 (5)	O5^xii^—O6—O5	134.5 (17)
O1^i^—Mg—B1^ii^	123.25 (4)	O5^xi^—O6—H5	104.1
O1^ii^—Mg—B1^ii^	121.16 (4)	O6^ix^—O6—H5	33.1
O1—Mg—B1^ii^	29.91 (4)	O6^xi^—O6—H5	103.9
B1—Mg—B1^ii^	114.20 (3)	O5^xii^—O6—H5	117.5
B1^i^—Mg—B1^ii^	114.20 (3)	O5—O6—H5	29.6
O2^i^—Mg—K	131.38 (3)	O5^xi^—O6—H6A	44.8
O2^ii^—Mg—K	131.38 (3)	O6^ix^—O6—H6A	101.0
O2—Mg—K	131.38 (3)	O6^xi^—O6—H6A	103.2
O1^i^—Mg—K	74.30 (3)	O5^xii^—O6—H6A	111.4
O1^ii^—Mg—K	74.30 (3)	O5—O6—H6A	111.4
O1—Mg—K	74.30 (3)	H5—O6—H6A	130.2
B1—Mg—K	104.19 (4)	O5^xi^—O6—H6B	149.2
B1^i^—Mg—K	104.19 (4)	O6^ix^—O6—H6B	122.2
B1^ii^—Mg—K	104.19 (4)	O6^xi^—O6—H6B	122.0
O3—K—O3^iii^	65.411 (16)	O5^xii^—O6—H6B	76.7
O3—K—O3^i^	114.589 (16)	O5—O6—H6B	78.5
O3^iii^—K—O3^i^	180.0	H5—O6—H6B	95.3
O3—K—O3^iv^	65.410 (16)	H6A—O6—H6B	104.5
O3^iii^—K—O3^iv^	114.590 (15)	O5^xi^—O6—H5^xi^	58.1 (15)
O3^i^—K—O3^iv^	65.410 (15)	O6^ix^—O6—H5^xi^	74 (3)
O3—K—O3^ii^	114.590 (16)	O6^xi^—O6—H5^xi^	28.4 (18)
O3^iii^—K—O3^ii^	65.410 (15)	O5^xii^—O6—H5^xi^	74 (3)
O3^i^—K—O3^ii^	114.590 (15)	O5—O6—H5^xi^	114 (3)
O3^iv^—K—O3^ii^	180.0	H5—O6—H5^xi^	87.3
O3^v^—K—O3^ii^	65.410 (16)	H6A—O6—H5^xi^	97.8
O3—K—Mg^v^	76.32 (2)	H6B—O6—H5^xi^	148.0
O3^iii^—K—Mg^v^	103.68 (2)	O3^xiii^—B1—O2	123.87 (12)
O3^i^—K—Mg^v^	76.32 (2)	O3^xiii^—B1—O1^i^	122.17 (12)
O3^iv^—K—Mg^v^	103.68 (2)	O2—B1—O1^i^	113.96 (12)
O3^v^—K—Mg^v^	103.68 (2)	O3^xiii^—B1—Mg	166.22 (10)
O3^ii^—K—Mg^v^	76.32 (2)	O2—B1—Mg	57.12 (7)
O3—K—Mg	103.68 (2)	O1^i^—B1—Mg	58.91 (7)
O3^iii^—K—Mg	76.32 (2)	O3^xiv^—B2—O2	112.28 (11)
O3^i^—K—Mg	103.68 (2)	O3^xiv^—B2—O1^xv^	109.48 (11)
O3^iv^—K—Mg	76.32 (2)	O2—B2—O1^xv^	110.35 (11)
O3^v^—K—Mg	76.32 (2)	O3^xiv^—B2—O4	109.12 (10)
O3^ii^—K—Mg	103.68 (2)	O2—B2—O4	107.66 (11)
Mg^v^—K—Mg	180.000 (17)	O1^xv^—B2—O4	107.83 (10)
			
O6^ix^—O5—O6—O5^xi^	−36 (3)	B1—O2—B2—O3^xiv^	21.1 (2)
O6^x^—O5—O6—O5^xi^	−94.9 (17)	Mg—O2—B2—O3^xiv^	−119.29 (10)
O6^x^—O5—O6—O6^ix^	−59 (2)	B1—O2—B2—O1^xv^	−101.35 (17)
O6^ix^—O5—O6—O6^xi^	52 (3)	Mg—O2—B2—O1^xv^	118.27 (10)
O6^x^—O5—O6—O6^xi^	−7 (5)	B1—O2—B2—O4	141.21 (14)
O6^ix^—O5—O6—O5^xii^	117 (3)	Mg—O2—B2—O4	0.84 (12)
O6^x^—O5—O6—O5^xii^	58 (4)	B2^i^—O4—B2—O3^xiv^	45.6 (2)
B2—O2—B1—O3^xiii^	16.3 (2)	B2^ii^—O4—B2—O3^xiv^	−162.41 (10)
Mg—O2—B1—O3^xiii^	163.52 (12)	B2^i^—O4—B2—O2	−76.54 (15)
B2—O2—B1—O1^i^	−163.39 (13)	B2^ii^—O4—B2—O2	75.49 (15)
Mg—O2—B1—O1^i^	−16.21 (12)	B2^i^—O4—B2—O1^xv^	164.40 (10)
B2—O2—B1—Mg	−147.18 (17)	B2^ii^—O4—B2—O1^xv^	−43.6 (2)

Symmetry codes: (i) *y*, *z*, *x*; (ii) *z*, *x*, *y*; (iii) −*y*+1, −*z*+1, −*x*+1; (iv) −*z*+1, −*x*+1, −*y*+1; (v) −*x*+1, −*y*+1, −*z*+1; (vi) *y*+1/2, *z*, −*x*+1/2; (vii) −*x*+1/2, *y*+1/2, *z*; (viii) −*y*+1/2, −*z*+1, *x*+1/2; (ix) −*y*+1/2, *z*−1/2, *x*; (x) −*z*+1, *x*−1/2, −*y*+1/2; (xi) *z*, −*x*+1/2, *y*+1/2; (xii) *y*+1/2, −*z*+1/2, −*x*+1; (xiii) −*x*+1/2, *y*−1/2, *z*; (xiv) *z*−1/2, −*x*+1/2, −*y*+1; (xv) −*z*+1/2, *x*−1/2, *y*.

### Hydrogen-bond geometry (Å, º)

**Table:**

*D*—H···*A*	*D*—H	H···*A*	*D*···*A*	*D*—H···*A*
O5—H5···O5^xii^	0.85	1.67	2.42 (3)	145
O6—H6*A*···O2^xii^	0.85	2.58	3.276 (15)	140
O6—H5···O6^ix^	0.65	0.74	1.20 (3)	119
O6—H5···O6^x^	0.65	1.23	1.84 (2)	158
O6—H5···O6^xvi^	0.65	1.82	2.19 (3)	118
O6—H5···O5^ix^	0.65	1.79	2.34 (3)	143
O6—H5···O5^x^	0.65	2.09	2.484 (19)	121
O6—H5···O5	0.65	0.85	1.35 (3)	128
O5—H5···O6^xii^	0.85	1.78	2.484 (19)	139
O5—H5···O6^xi^	0.85	1.49	2.34 (3)	179
O5—H5···O6^xvi^	0.85	1.82	2.30 (2)	114
O5—H5···O5^xii^	0.85	1.67	2.42 (3)	145
O5—H5···O5^xi^	0.85	1.28	1.88 (3)	122
O5—H5···O5^xvi^	0.85	2.30	3.06 (3)	150

Symmetry codes: (ix) −*y*+1/2, *z*−1/2, *x*; (x) −*z*+1, *x*−1/2, −*y*+1/2; (xi) *z*, −*x*+1/2, *y*+1/2; (xii) *y*+1/2, −*z*+1/2, −*x*+1; (xvi) −*x*+1, −*y*, −*z*+1.
